# Antibodies in the breast milk of a maternal woman with COVID-19

**DOI:** 10.1080/22221751.2020.1780952

**Published:** 2020-07-03

**Authors:** Yunzhu Dong, Xiangyang Chi, Huang Hai, Liangliang Sun, Mengyao Zhang, Wei-Fen Xie, Wei Chen

**Affiliations:** a Beijing Institute of Biotechnology, Beijing, People’s Republic of China; b Changzheng Hospital, Second Military Medical University, Shanghai, People’s Republic of China

**Keywords:** COVID-19, maternal woman, breastmilk, IgG, IgA

## Abstract

A maternal woman was positive for SARS-CoV-2 tested in throat swabs but negative tested in other body fluids, and she had IgG and IgA detected in breast milk. Her infant negative for SARS-CoV-2 at birth had elevated IgG in serum but quickly decayed. These findings suggest that breastfeeding might have the potential benefit to the neonates.

The ongoing novel coronavirus (COVID-19) outbreak caused by severe acute respiratory syndrome coronavirus 2 (SARS-CoV-2) has posed a global public health concern [1]. The number of pregnant women and neonate with COVID-19 is also on the rise [2, 3]. A recent study reported a newborn with elevated IgM antibodies to SARS-CoV-2 born to a mother with COVID-19 [4]. Vertical transmission of SARS-Cov-2 and transplacental transmission of antibodies have been proposed with controversial opinions [5–7]. Although clinical and laboratory characteristics, and outcomes of pregnant women with COVID-19 have been reported [4], there are no continuously monitored data about the viral loads in several body fluids of the maternal women that would bring potential risks of SARS-CoV-2 infection to neonates [8]. Here, we follow up the viral loads and antibody titers of SARS-Cov-2 in a maternal woman and the neonate since be hospitalized to discharged.

On February 26, 2020, a 33-year-old primiparous woman (38 weeks 2 days of gestation with irregular lower abdominal pain with vaginal fluid for 6 h) suffering from cough and chest tightness 2 weeks ago was admitted to hospital for childbirth. Chest radiography showed patchy ground-glass opacities in the periphery of left lung (Figure 1, panel A), and a throat swab was positive for SARS-CoV-2 through reverse-transcription real-time polymerase chain reaction (RT–PCR) at admission (Figure 1, panel B). Laboratory test abnormalities included the decrease of lymphocyte percentage (15.6%), increase of neutrophil percentage (80%), and elevated serum levels of hepatic enzymes (alanine aminotransferase 90 U/L, aspartate aminotransferase 81.8 U/L; Supplementary table). The maternal woman was considered as an ordinary case base on mild symptoms and radiologic imaging.

**Figure 1.**
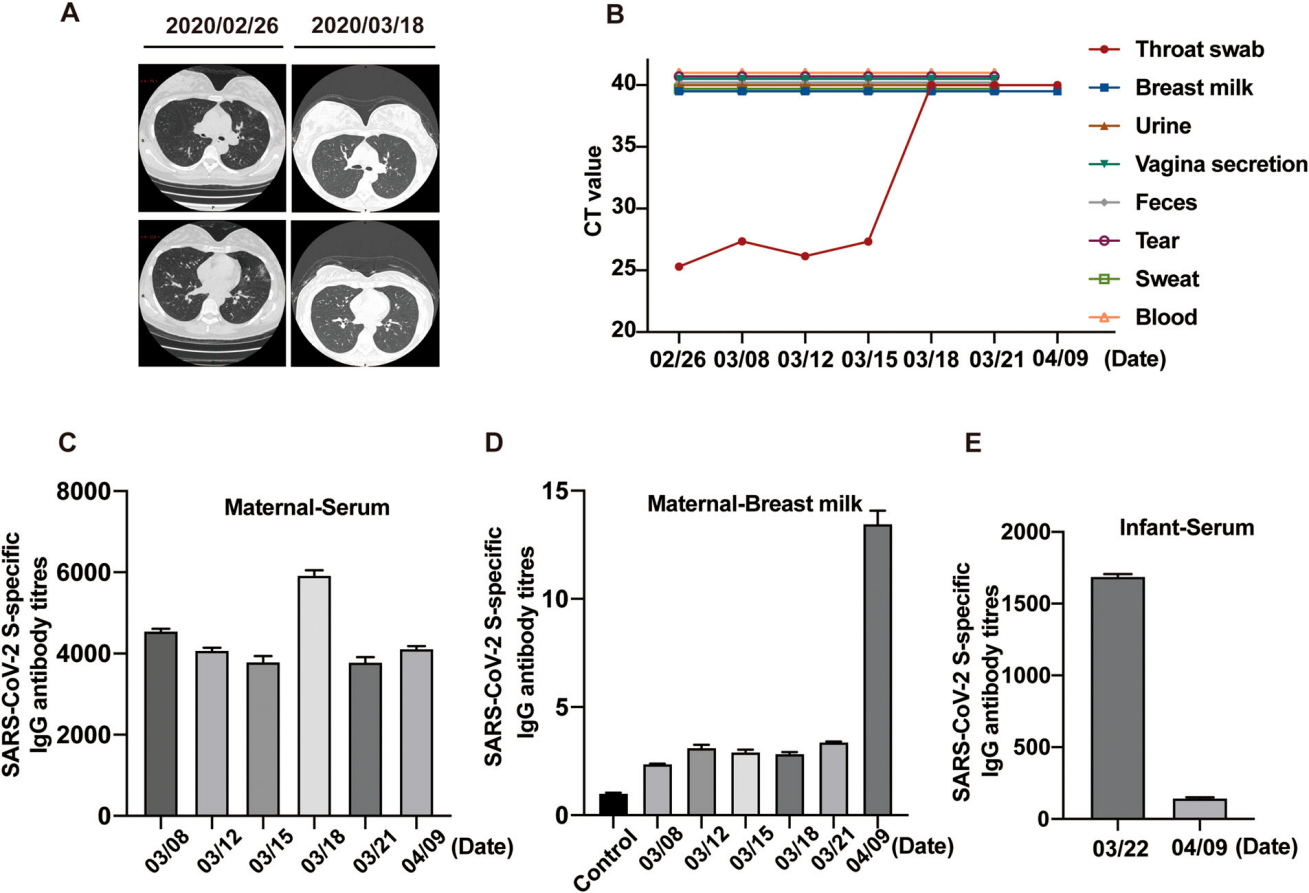
Detection of SARS-CoV-2 viral loads and IgG antibody in samples from the maternal woman and neonate. (A) Chest radiography of the maternal woman on February 26 and March 18. (B) Viral loads in samples collected from the maternal woman. (C) Titers of IgG antibody to SARS-CoV-2 in the maternal woman’s serums determined using ELISA. Data were shown as mean ± SD of three duplicates. (D) Titers of IgG antibody to SARS-CoV-2 in maternal woman’s breast milk determined using ELISA. Data were shown as mean ± SD of three duplicates. (E) Titers of IgG antibody to SARS-CoV-2 in the neonate’s serums determined using ELISA. Data were shown as mean ± SD of three duplicates.

Due to the pregnancy, there was neither antiviral nor antibiotic treatment for the patient. The woman gave birth in a negative-pressure operating room. All persons in the room wore protective suits and the maternal woman wear an N95 mask immediately after labor. The infant girl was delivered by Left Occiput Anterior (LOA) in isolation delivery room and quarantined in the neonatal intensive care unit (ICU). The infant’s birth weight was 2950 g, and Apgar scores were 9 at 1 min and 10 at 5 min. An oropharyngeal swab specimen, obtained immediately after she was taken from the uterus, showed a negative result for the detection of SARS-CoV-2 RNA. The infant was then sent to the negative-pressure ward. After delivery, the woman was transferred to the ICU isolation ward to continue treatment with anti-infective medication (Azithromycin and Ornidazole). On March 7, the woman was transferred to Guanggu Branch of Hubei Maternal and Child Health Hospital, a designated hospital for COVID-19 treatment in Wuhan city.

With the use of RT–PCR assays, the mother’s throat swabs were continuously positive, and the CT value remains low for SARS-CoV-2 by RT–PCR at March 8, 12 and 15, and turned negative since March 18, while all samples of breast milk, urine, vaginal secretion, feces, tear, sweat and blood serially collected during the same period were negative (Figure 1, panel B). Through Enzyme-linked immunosorbent assays (ELISAs) using SARS-CoV-2 spike protein as antigen, the titers of IgG antibody to SARS-CoV-2 in serum were 4509.5, 4025.4, 3683.6, 5838.6, 3690.1, respectively (Figure 1, panel C). The titers of IgG antibody in breast milk were 2.34, 3.02, 2.84, 2.79, and 3.35, respectively, when three SARS-CoV-2 negative maternal woman’s breast milk were tested as control (mean titer 0.98) (Figure 1, panel D). Breastfeeding protects infants against infections mainly via secretory IgA (SIgA) antibodies. In the early stages of lactation, IgA, anti-inflammatory factors and, more likely, immunologically active cells provide additional support for the immature immune system of the neonate. Here we determined the antibody to SARS-CoV-2 in breast milk by ELISA. The titers of IgA antibody in breast milk were 2.18, 2.23, 2.37 and 2.69, respectively, when three SARS-CoV-2 negative maternal woman’s breast milk was tested as control (Mean titer 0.9) (Supplementary figure).

On March 22, the mother was discharged, with her abnormal chest radiography and laboratory test findings recovered to normal, and the neonate had a negative result for detection of SARS-CoV-2 RNA in the throat swab and IgG titer of 1675.5 in serum, which was lower than her mother (Figure 1). On April 9, the mother’s IgG antibody remained a titer of 4066.6 in serum and elevated to 13.9 in breast milk, but the neonate’s IgG turned negative (Figure, panel D and E). And the titer of IgA antibody was slightly elevated to 4.01 in breast milk (Supplementary figure).

Although the SARS-CoV-2 has been detected in breast milk in recent publication [9], in our study there is no detection of SARS-CoV-2 in the mother’s body fluids serially collected after delivery when her throat swabs showing positive results for SARS-CoV-2, especially in breast milk. The neonate had negative result for SARS-CoV-2 RNA at the birth and her IgG antibody to SARS-CoV-2 was observed only within one and half month after birth, indicating the placenta transmission of COVID antibody. The mother’s IgG antibody usually remains in neonate for more than 6 months after birth. Infants incapable of producing immunoglobulins are protected by maternal antibodies for up to 12 months after birth [10], however, our follow-up identified that IgG to SARS-CoV-2 in the neonate maintained less than one and half month, suggesting the potential risk for subsequent COVID-19 in neonates. More importantly, the IgG and IgA antibodies were detected in breast milk indicate the potential immune protection for the neonates. Nonetheless, data from large-scale cohort are warranted.
